# Feasibility of a Mental Health App Intervention for Emergency Service Workers and Volunteers: Single-Arm Pilot Study

**DOI:** 10.2196/50995

**Published:** 2025-07-28

**Authors:** Denise Meuldijk, Mark Deady, Daniel A J Collins, Douglas O Williams, Richard A Bryant, Samuel B Harvey

**Affiliations:** 1 Black Dog Institute, Faculty of Medicine and Health, University of New South Wales, High St, Kensington, Sydney, NSW 2031, Australia, 61 02 9065 9103; 2 School of Psychology, University of New South Wales, Kensington, 2052, Australia

**Keywords:** digital mental health, emergency service workers, high risk professions, prevention, psychological distress, smartphone applications, user-engagement

## Abstract

**Background:**

Emergency service workers are at an elevated risk for stressor-related mental health (MH) issues, such as anxiety, depression, and posttraumatic stress disorder. Barriers to help-seeking are widespread across this sector, necessitating interventions tailored to the unique needs of this population. Build Back Better is a smartphone-based intervention designed to provide evidence-based strategies for the prevention of anxiety, depression, and posttraumatic stress disorder among emergency service workers.

**Objective:**

This study aimed to evaluate the usability, acceptability, feasibility, and preliminary effectiveness of the Build Back Better app among emergency service workers.

**Methods:**

A single-group (N=67), 1-month pilot study assessing the impact of the Build Back Better app on MH outcomes, including general distress, anxiety, depression, and traumatic stress coping, was undertaken with emergency service workers. Participants completed baseline and 1-month follow-up assessments using the Kessler Psychological Distress Scale, 9-item Patient Health Questionnaire, 7-item Generalized Anxiety Disorder, World Health Organization Well-Being Index, and the Trauma Coping Self-Efficacy Scale. The app’s usability and acceptability were also evaluated through participant feedback and usage data.

**Results:**

Of the 71 participants enrolled, 67 completed the baseline assessment and downloaded the app, with 33 participants providing follow-up data. The mean age of participants was 44.73 (SD 11.4) years, and 64% (n=43) were male. The majority of respondents rated the app quality as very high (n=27, 79%), felt that the app was easy to use (n=20, 61%), easily understood (n=18, 55%), improved their mental fitness (n=27, 80%), and would recommend the app to others (n=20, 61%). Encouraging trends toward improvement were found across symptom and well-being outcomes; however, these trends were not significant: general distress (*t*_32_=0.65, *P*=.52), depression (*t*_32_=0.75, *P*=.46), anxiety (*t*_32_=1.08, *P*=.29), or traumatic stress coping (*t*_32_=−0.27, *P*=.79), with effect sizes ≤0.2, likely due to the small sample size.

**Conclusions:**

The Build Back Better app demonstrated satisfactory levels of usability and acceptability. While the pilot study showed encouraging trends toward improved MH, further research with a larger sample size is needed to determine its efficacy. Participants furthermore suggested improvements in app navigation and content clarity, emphasizing the need for a more intuitive user experience. Given the positive feedback and improvement in MH outcomes, a larger-scale efficacy trial is warranted to further assess the app’s potential for MH support in this high-risk population.

## Introduction

### Background

Emergency service workers and volunteers are those workers (both paid and volunteer) who attend scenes of emergencies and disasters to minimize risk to community safety and security. These include (but are not limited to) police, paramedics, firefighters, rescue, and emergency service personnel. They perform an indispensable function in the community; however, the nature of this work can come at a cost to their mental health (MH) and well-being [1]. These workers are exposed to elevated rates of stress, trauma, and adversity on a regular basis, putting them at increased risk for a range of MH conditions, including, but not limited to, depression, anxiety, drug and alcohol-related disorders, and posttraumatic stress disorder (PTSD) [2-5].

The question of what measures may be able to enhance or protect MH in emergency service workers is garnering growing interest internationally. A recent survey by Kyron et al [6] among 14,868 Australian ambulance, fire and rescue, police, and state emergency services employees highlights the range of MH and well-being conditions associated with work in the emergency services sector. While 10% of employees screened positive for probable PTSD, 30% had low well-being, and 30% had high or very high psychological distress, suggesting that depression and anxiety, as well as PTSD, are likely to be common MH issues for emergency service workers. Similar results were found in a systematic review of 27 international studies [7] reporting on 30,878 ambulance personnel, which found estimated prevalence rates of 11% for PTSD, 15% for depression, 15% for anxiety, and 27% for general psychological distress. In a study of 5813 Canadian safety personnel (correctional workers, dispatchers, firefighters, paramedics, and police officers), Carleton et al [8] found that 44.5% screened positive for clinically significant symptoms of one or more diagnosable mental disorders, approximately 4 times higher than rates of diagnosis for the general population. Of concern, these rates were noted to be higher than those reported in previously conducted studies in the area, suggesting that mental disorders may be increasing among emergency service workers [8-10].

Despite the high prevalence of MH symptoms, significant barriers continue to hinder and delay help-seeking among emergency service workers, including stigma, perceived unavailability of resources, the need for self-reliance in solving problems, and fear about the intervention or treatment [11]. Emergency service workers’ work constraints are also different from many other professions, which can influence seeking help for MH problems even more. For example, many emergency service workers are often “on duty” for 24 to 72 hours, thereby having to respond to unpredictable emergencies, which supports the importance of tailored interventions, accessible at any time [12,13]. Their culture is different as well; when emergency service workers return home, they are expected to “function” in daily living, such as work and family life, which increases the likelihood that any MH symptoms may be ignored. Finally, emergency service workers are reluctant to approach mainstream MH services because of fear that awareness by their organization of MH problems may negatively impact certain privileges, such as promotion, deployment to select units, or the right to carry a firearm, and so on. Taken together, these factors point to the importance of considering emergency service workers’ perspectives and needs when devising MH interventions and the necessity of developing novel MH approaches with widespread reach.

Mobile phone–based health initiatives have become an increasingly popular way to provide MH care and support to individuals [14]. The increased use of technology, alongside the widespread treatment gap, has led researchers to explore the use of digital platforms for MH engagement. Smartphone apps are considered a suitable prevention approach, since they have the potential to deliver effective psychological interventions via frequent brief exchanges, offering a unique opportunity for disorder prevention and symptom management [15,16]. The multiple features of mobile phones furthermore offer great opportunities for the delivery of health promotion interventions in different formats, thereby having the potential to overcome traditional service barriers [17], especially in difficult-to-access populations, such as emergency service workers. Apps enable flexible user engagement, allowing individuals to access interventions at their convenience, in private, and at a time and location of choice, thereby facilitating real-time monitoring of mood and behavior [18]. Meta-analyses of randomized controlled trials have shown promising results for evidence-based MH apps in reducing depressive symptoms, stress, anxiety, and substance use [19,20]. Moreover, MH applications can successfully use persuasive strategies, supporting people with MH issues to adopt healthy lifestyles and improving user-oriented behavior changes [17,21].

Prior to efficacy testing, a critical aspect in the development of mobile phone apps is exploring the acceptability and feasibility of the interventions [22]. Engaging the target audience in the process of development is essential for developing optimum treatments in specific settings and is likely to contribute to the wider acceptability and utility of digital MH interventions [22,23].

### Objectives

This pilot study aims to explore the use, acceptability, and preferences of emergency service workers regarding potential uptake of a smartphone-based MH intervention designed to provide evidence-based MH prevention strategies, the Build Back Better app. As well as assessing acceptability and feasibility, this study also aims to assess the impact of the Build Back Better app on depression, anxiety, well-being, and other symptoms of common mental disorders.

## Methods

### Study Design and Setting

The study was conducted with emergency service workers from 5 different emergency and rescue services organizations across Australia. Participants were recruited via organizational advertisements, which included QR codes or links to the study website. Participants were recruited to test usability, acceptability, feasibility, and preliminary MH effects of a smartphone-based MH intervention: the Build Back Better app. Data were collected via pre- and postintervention questionnaires and in-app data collection, with participants using the app remotely. Recruitment started on August 16, 2021, and was finalized on December 18, 2021. Data collection of the trial was completed by January 2022.

### Ethical Considerations

The study was granted ethical approval by the University of New South Wales Human Research Ethics Committee (HC210399). Informed consent was collected electronically before participation. This approval covers secondary analysis without additional consent. Participants were compensated with an Aus $40 (US $26) gift voucher for completing the post-assessment and qualitative interview. Alternatively, participants could opt for an Aus $40 (US $26) donation to a charity if their organization had gift restrictions. The pilot study was registered in the Australian New Zealand Clinical Trials Registry (ACTRN12621001006831p), and the app was notified with the Therapeutic Goods Administration through the Clinical Trial Notification scheme (CT-2021-CTN-02958‐1). Study data were deidentified to ensure confidentiality and participant privacy.

### Participants

#### Eligibility

Eligible participants were Australian residents, aged 18 years and older, currently working, or previously (ie, in the last 2 years), as an emergency service worker (volunteer or paid). Retired emergency service workers (ie, emergency service workers who have worked in the last 2 years) were also deemed eligible to participate due to both the delayed onset of many trauma-related symptoms and the fact that such symptoms may have been implicated in a decision to retire [1]. Other inclusion criteria included the ability to speak and understand English, willingness and ability to provide informed consent, and access to an Android or iOS smartphone. Participants were also required to have a current email address or be willing to obtain one.

#### Recruitment

Participants were recruited via organizational advertisements calling for emergency service workers to test a new MH app. QR codes or links on the advertisements directed interested individuals to the study website, which included more detailed information about the study procedures and inclusion criteria.

All interested participants were required to provide informed consent electronically. Baseline assessment occurred immediately after participant consent. Upon completion of the baseline assessment, participants were prompted to download Build Back Better from either the App Store (for iPhone users) or the Google Play Store (for Android users) and use the app for approximately 30 days. Postassessment occurred 1 month after the completion of the baseline assessment. After the completion of the postassessment surveys, participants were invited to participate in a qualitative interview to ascertain in-depth information on app use and user preferences (also see Qualitative Data Collection section for details). See Table 1 for an overview of the different measures administered at baseline and 1-month follow-up. All assessments were administered online using Black Dog Institute’s Online Research Platform.

**Table 1.** Overview of study measures and data collection time points.

Instrument	Domain	Time points		
		Baseline	Postassessment^a^	In-app data collection^b^
Demographic Inventory	Demographics	✓		
Kessler Psychological Distress Scale	Psychological distress	✓	✓	
9-item Patient Health Questionnaire	Depression	✓	✓	
7-item General Anxiety Disorder	Anxiety	✓	✓	
5-item World Health Organization Well-Being Index	Well-being	✓	✓	
Trauma Coping Self-Efficacy	Trauma coping	✓	✓	
App engagement (survey and qualitative interviews)	App feasibility and acceptability		✓	
Objective app engagement outcomes	App feasibility and acceptability			✓

a Postassessment administered 1 month after baseline assessment.

b In-app data collection throughout the entire duration of study (ie, from baseline to 1-month follow-up).

### Intervention

**Figure 1.**
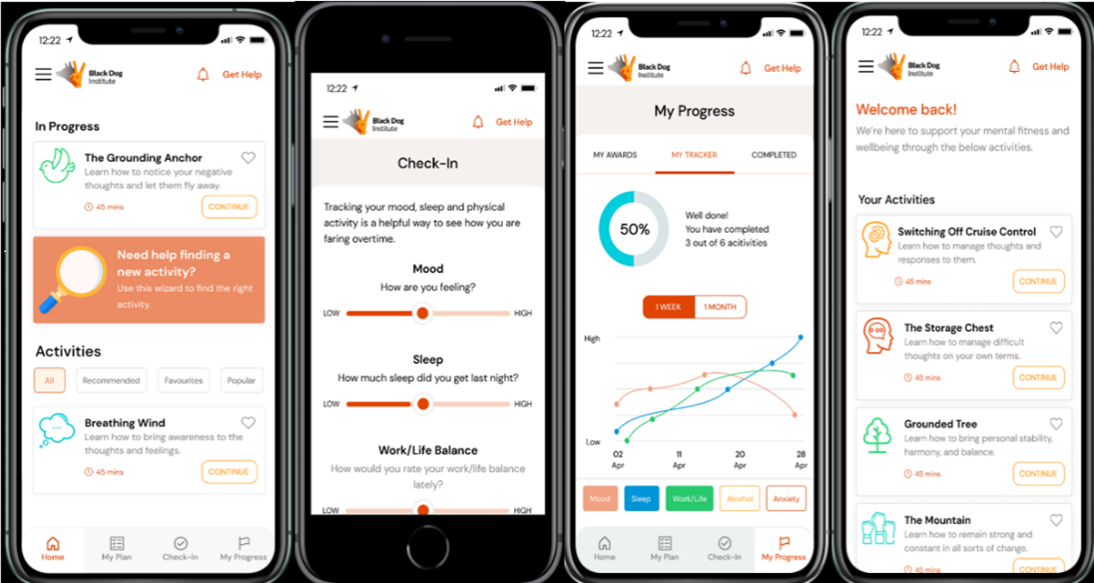
Screenshots of key features of the Build Back Better app.

Build Back Better was developed by a multidisciplinary team with content adapted from a previously developed app, HeadGear*,* designed to prevent depression and anxiety and improve well-being among workers in male-dominated industries [18,22]. A randomized controlled trial of HeadGear with a large sample of workers showed that the app was effective in the prevention of depression and anxiety, as well as improving a range of MH and work-related outcomes [24,25]. While many other MH apps offer generic advice and often fail to offer personalized care, the Build Back Better app was carefully developed through extensive collaboration with clinical psychologists, psychiatrists, IT professionals, user experience and app design experts, and, most importantly, emergency service workers themselves. This approach ensures that the app content and design are tailored to the specific needs of emergency service workers and their families. By integrating trauma-focused content alongside strategies for addressing depression and anxiety, the app tailors its support based on the preferences and needs expressed by emergency service workers, leading to better, more effective outcomes [26]. Personalized changes to the app visual presentation and design included a more gender-neutral look and feel (achieved through color scheme, imagery, language, and theme) and streamlined features to promote use in rural populations with unreliable internet access or phone service (ie, videos or activities that can be accessed without an internet connection) and those with limited mobile phone digital literacy. In addition, changes ensuring that the app’s structure would be suitable for those performing shift work were included, by allowing day-to-day use at all times, and whenever desired. For example, whereas previous apps developed by the research team [18,22] present app content in a linear form to be completed on consecutive days, the Build Back Better app content is tailored to the individual needs, via an upfront needs analysis, and through an in-app search function, maximizing user control and autonomy [27]. As it is suggested that this target population typically uses apps in their own time without clinical oversight, they must be intrinsically motivated to engage with the app [26,28]. Therefore, use flexibility was maximized by allowing the user to complete tasks freely within the app, according to their priorities and preferences. To further target the needs of emergency service workers, trauma-informed content was developed specifically for the app through consultation with clinical psychologists working primarily with emergency service workers. This clinical content incorporates thought monitoring or challenging, behavioral activation, problem solving, mindfulness, elements of positive psychology (eg, gratitude), and techniques to enhance healthy coping behaviors. Other components of the Build Back Better app include a tracker for monitoring mood, physical activity, sleep, eating habits, and work and life balance, and a variety of breathing and grounding exercises, based on the expressed preferences of emergency service workers. See Figure 1 for screenshots of the Build Back Better app.

### Data Collection

At baseline, participants were asked questions regarding demographics, MH illness in the previous 2 years, help-seeking behaviors for an MH problem in the previous month (from any source including health professionals, telephone or online support services, family, partner, or friends), and whether they were currently taking medication for an MH issue.

App usability, feasibility, and acceptability were measured using questions adapted from the Mobile Application Rating Scale (MARS; Stoyanov et al [29]) at postassessment, including questions pertaining to current smartphone use, potential future use, and preferences for design and content. The MARS is a simple, objective, and reliable tool for classifying and assessing the quality of mobile health apps [29]. The app quality criteria were clustered within the engagement, functionality, aesthetics, information quality, and subjective quality categories, from which 7 individual MARS items were included. Each MARS item used a 5-point scale (“not at all” to “completely” or “definitely”). In addition, 4 open-ended questions were administered, allowing the user to express their opinion about the app, in their own words. In addition, those agreeing to take part in the qualitative semistructured interview were asked a series of open-ended questions pertaining broadly to functionality, engagement, aesthetics, information exchange, and the user’s overall experience with the app to further explore app usability and acceptance. To monitor app usage, in-app data was automatically collected for the duration of the study (ie, from baseline to 1-month follow-up), such as number of times app opened, number of activities accessed, number of activities completed, and total time spent in app.

Psychological distress was measured using the Kessler Psychological Distress Scale (K10) [30]. The K10 is a 10-item measure of psychological distress over the past 4 weeks and is intended to yield a global measure of distress based on questions about anxiety and depressive symptoms. The K10 uses a 5-value response option for each question, which can be scored from 5 (“all of the time”) through 1 (“none of the time”). The total K10 score is based on the sum of the answers given to the 10 questions and was used to indicate psychological distress across all study time points. The maximum score is 50, indicating severe distress; the minimum score is 10, indicating no distress.

Depressive symptoms were measured using the 9-item Patient Health Questionnaire (PHQ-9) ([31]. The PHQ-9 is a reliable and valid 9-item self-report tool designed to assess depression severity. The items duplicate the 9 diagnostic criteria for major depressive disorder covered in the *Diagnostic and Statistical Manual of Mental Disorders, Fourth Edition* (DSM-IV). The PHQ-9 asks how often participants have been bothered by problems in the past 2 weeks. Each item is scored on a 4-point Likert scale, ranging from 0 (“not at all”) to 3 (“nearly every day”). Items are summed to provide a total score (ranging from 0 to 27), which was used as a marker of depressive symptom severity across all study time points. Scores ≤4 suggest minimal depression, which may not require treatment, whereas scores of ≥10 are indicative of depression.

Anxiety symptoms were measured using the 7-item Generalized Anxiety Disorder (GAD-7) [32], a reliable and valid 7-item measure of generalized anxiety symptoms [31]. GAD-7 scores can range from 0 to 27, with 5, 10, and 15 representing cutoffs for mild, moderate, and severe levels of anxiety, respectively. A score of 10 or greater on the GAD-7 represents a reasonable cut point for identifying cases of GAD.

Participant well-being was measured using the 5-item World Health Organization Well-Being Index (WHO-5) [33]. The WHO-5 is a short, self-administered measure of well-being over the last 2 weeks. It consists of 5 positively worded items that are rated on a 6-point Likert scale, ranging from 0 (“at no time”) to 5 (“all of the time”). The raw scores were transformed to a score from 0 to 100, with lower scores indicating worse well-being. A score of ≤50 indicates poor well-being and suggests further investigation into possible symptoms of depression. A score of 28 or below is indicative of depression.

Perceived self-efficacy for coping with challenges and threats was measured using the 9-item Trauma Coping Self-Efficacy Scale (CSE-T) [34]. Participants were asked to rate their capability to handle a series of posttraumatic situations (eg, “Deal with my emotions [anger, sadness, depression, anxiety] since the traumatic event,” “Manage distressing dreams or images about the traumatic experience”), on a 7-point scale ranging from 1 (“not at all capable”) to 7 (“totally capable”). Total CSE-T scores (ranging between 1 and 63) were created by summing the item ratings across all time points to analyze whether CSE-T increased as participants continued to use the application (ie, reflecting improvement in self-efficacy perceptions).

### Qualitative Data Collection

After the completion of the postassessment surveys, participants were invited to participate in a qualitative interview to ascertain in-depth information on app use and user preferences. Interviews (approximately 1 hour) occurred within 2 weeks of postassessment and were conducted virtually (ie, using telephone or videoconference).

Participation in postassessment surveys and the qualitative interviews was entirely voluntary and did not affect the use of the app. Users could engage with the app as they wish, regardless of their involvement in the postassessment surveys and qualitative interviews. Participants were, however, offered an Aus $40 (US $26) gift voucher as time reimbursement upon completion of the postassessments and the qualitative interview; if this was against the gift policy of their organization, they could select an Aus $40 (US $26) donation to a charity of their choice.

### Sample Size

Based on earlier comparable (pilot) studies using eHealth interventions [22,35,36], it was estimated that a sample size of 63 was required to detect a small within-group effect difference (Cohen *d*=0.3) with 80% power and a 0.05 significance level. With the expectation that 30% (n=20) of participants will be ineligible, lost to follow-up, drop-outs, or noncompliant, at least 90 participants would need to be enrolled.

### Data Analysis

Analyses were both quantitative and qualitative. Study investigators examined descriptive characteristics of the study sample and overall feasibility and acceptability of the smartphone app within the pilot study. Potential efficacy of the app in reducing psychological distress was assessed by evaluating changes in clinical outcomes after 1 month of use in terms of both statistically significant changes as well as effect size estimates. Changes in participants’ well-being and MH levels from baseline to posttest were examined using paired sample *t* tests. Qualitative data collected through semistructured interviews were examined to understand user needs, experience, satisfaction, and expectations of the app. Study investigators extracted themes based on participants' responses to understand participants’ acceptability of the study design and overall experience with the smartphone app.

## Results

### Participants Characteristics

**Figure 2.**
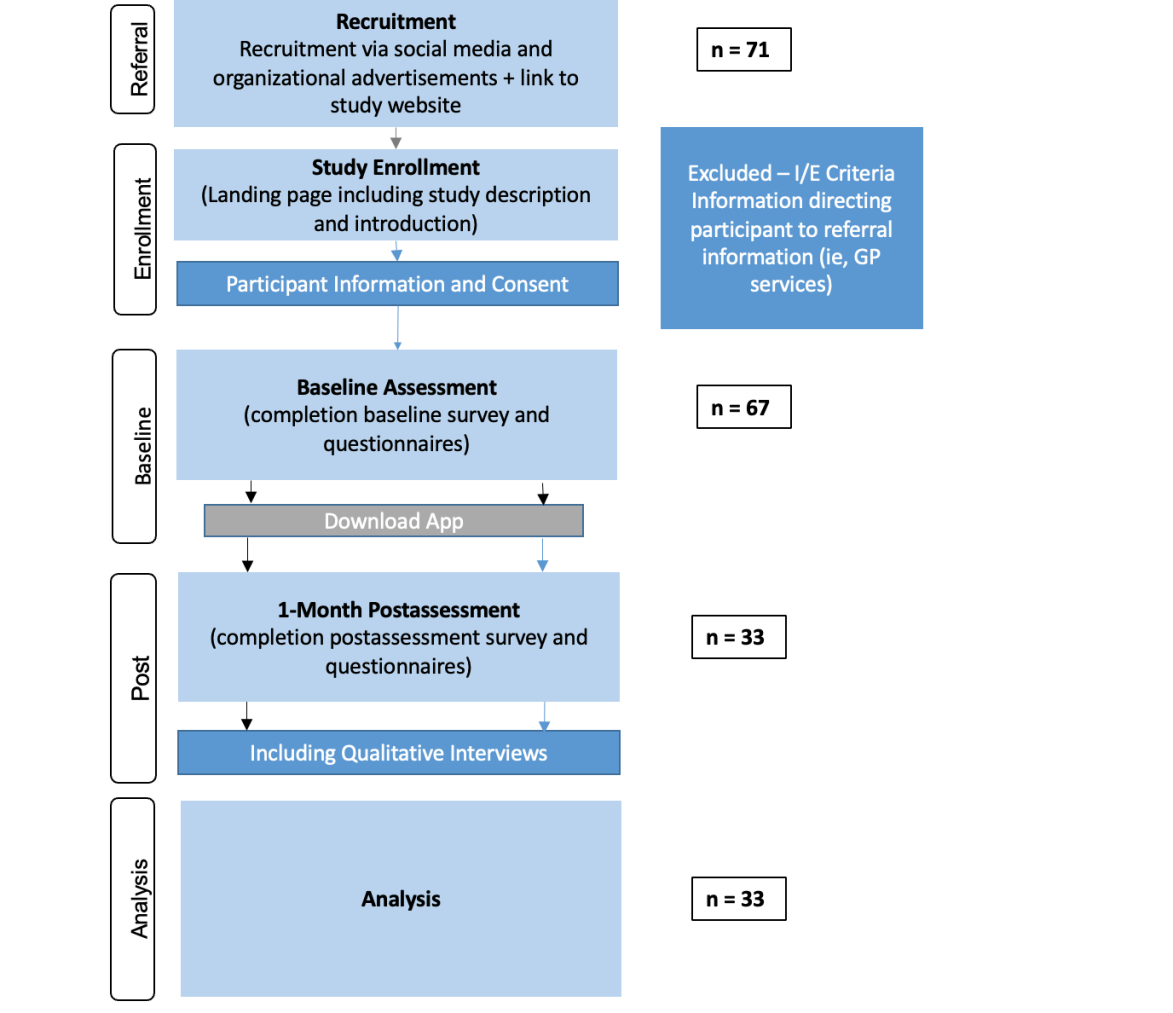
CONSORT (Consolidated Standards of Reporting Trials) flow diagram illustrating the study procedures. GP: general practitioner; I/E: inclusion/exclusion.

A total of 71 participants enrolled in the study, of which 67 both completed the baseline assessment and downloaded the app (see Figure 2 for the CONSORT flow diagram of this single-arm study). The baseline characteristics of the sample (N=67) are presented in Table 2. The mean age of the participants was 44.73 (SD 11.40; range 29‐66) years, with the majority being male (n=43, 64%). Participants included firefighters, disaster response, surf lifesaving, police, and correctional services across 4 Australian states: New South Wales (n=12), South Australia (n=5), Tasmania (n=17), and Western Australia (n=33). The sample was largely drawn from major cities (>60%), and approximately half were volunteer emergency service workers (48%). More than 80% reported an episode of poor MH lasting at least 1 month within the last 4 weeks, with 44% having sought some form of MH support in the previous month (eg, from a general practitioner, MH professional, telephone or online support services, family, partner, or friends), and 16% (n=11) currently using medication for an MH issue.

Although the majority of the sample (n=41, 62%) reported experiencing no current distress (ie, a score under 20) on the K10, more than a quarter (n=18, 27%) reported either moderate or severe distress (ie, a score of 25 or higher) on the K10 at baseline (refer to Table 2). Around half of the participants (n=35, 52.2%) reported minimal anxiety symptoms (ie, a score of 4 or under), whereas 45% (n=30) scored between 15 and 24 on the GAD-7, indicative of mild to moderate anxiety symptoms. A total of 3% (n=2) of the participants reported severe anxiety symptoms (ie, a score >15 on the GAD-7). Prevalence of depression symptoms (ie, a score of ≥10 on the PHQ-9) was reported in 24% (n=16), and minimal to mild depression severity (ie, a score of <10 on the PHQ-9) was reported in 76% (n=51) of the participants.

Of the 67 app users who completed the baseline assessment, 51% (n=34) failed to complete the follow-up questionnaire, resulting in complete follow-up data for 33 users. The only significant difference between postcompleters and noncompleters was sex, with males being less likely to complete the postassessment. Noncompleters reported worse MH on all measures at baseline, although none of these differences reached statistical significance.

**Table 2.** Sociodemographic and clinical characteristics of study participants.

Demographics	Values
Age (years)	
Mean (SD)	44.73 (11.4)
Range	29-66
Male, n (%)	43 (64.2)
Highest level of education, n (%)	
University	13 (19.4)
Postgraduate	9 (13.4)
Non-university diploma/college	14 (20.9)
Trade or other certificate	17 (25.4)
Year 12 certificate	11 (16.4)
Year 10 certificate	2 (3)
Other	1 (1.5)
State, n (%)	
New South Wales	12 (17.9)
South Australia	5 (7.5)
Tasmania	17 (25.4)
Western Australia	33 (49.3)
Agency, n (%)	
Airservices Australia^a^	2 (3)
DBCA^b^	3 (4.5)
DFES^c^	30 (44.8)
DPFEM^d^	16 (23.9)
RFS^e^	4 (6)
SAFECOM^f^	5 (7.5)
SLSA^g^	7 (10.4)
Emergency service worker - volunteer, n (%)	32 (47.8)
Location, n (%)	
Metropolitan	44 (65.7)
Rural	11 (16.4)
Remote	12 (17.9)
General distress (K10)^h^, n (%)	
No distress	41 (61.2)
Mild distress	8 (11.9)
Moderate distress	9 (13.4)
Severe distress	9 (13.4)

a Airservices: Aviation Rescue Fire Fighting Service - Airservices Australia (New South Wales).

b DBCA: Department of Biodiversity, Conservation and Attractions (Western Australia).

c DFES: Department of Fire and Emergency Services (Western Australia).

d DPFEM: Department of Police, Fire & Emergency Management (Tasmania).

e RFS: New South Wales Rural Fire Service (New South Wales).

f SAFECOM: South Australian Fire and Emergency Services Commission (South Australia).

g SLSA: Surf Life Saving Australia (New South Wales).

h K10: Kessler Psychological Distress Scale.

### Intervention Delivery

The frequency of app usage ranged from once to 22 days (mean 5.5, SD 5.62), with usage averaging 25 minutes per session. In-app activities related to trauma, recognizing poor MH, identifying thought patterns, breathing, and mindfulness exercises were the most accessed by users. The daily check-in feature (allowing users to rate their mood or behaviors and displaying a weekly and monthly graph of their past mood check-ins) was the most popular app feature, being accessed by 43% (n=15) of participants during the trial period. On average, after opening an activity within the app, 67% (n=22) of participants completed the full activity content.

**Figure 3.**
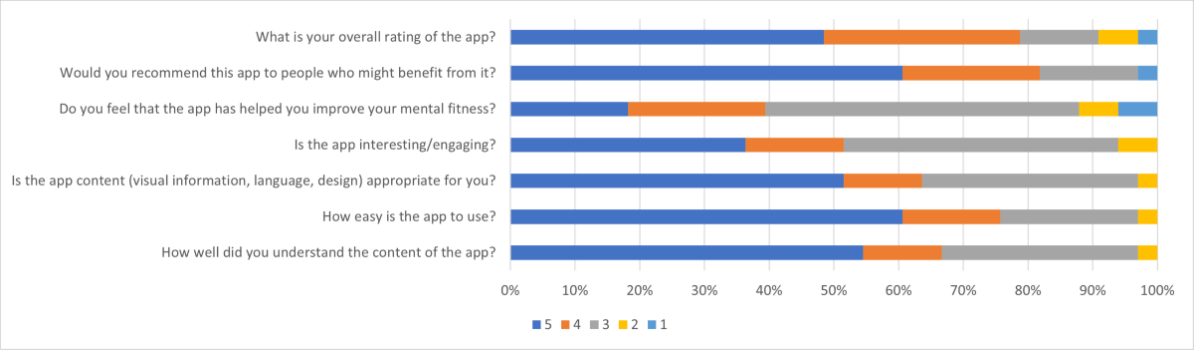
Participant ratings of acceptability variables using a 5-star rating scale. The specific meanings of the 5-star rating scale labels may vary depending on the item, but in general, 5 points represent excellent or outstanding performance, while 1 point represents a poor experience or performance.

Figure 3 presents the results for the different acceptability variables. At 1-month follow-up, most of the participants (n=29, 88%) felt that the Build Back Better app had at least ‘‘somewhat” improved their mental fitness. The majority of participants (n=27, 79%) rated the overall quality of the app as “very high” or “high,” with a mean score of 4 out of 5. Although more than half of the participants (n=18, 55%) claimed that they “completely” understood the app content*,* 30% (n=10) indicated the content of the app was “somewhat well” understood. The majority of participants (n=20, 61%) reported that using the app was very easy (with a mean rating of 4 out of 5), and that they would “definitely” be willing to recommend the app to others. Around half (n=17, 52%) indicated that the app content (visual information, language, and design) was appropriate, whereas one-third (n=11, 33%) indicated the app content was “somewhat” appropriate for them. In terms of app interest and engagement, the participant’s response was somewhat mixed. A total of 36% (n=12) of the participants indicated the app was “completely or highly” engaging, whereas 42% (n=14) of participants rated the app as “somewhat interesting.”

### Qualitative Data

Open feedback on the app from 33 participants who completed their 1-month follow-up assessment was generally positive and encouraging:

*It is a great app, a very useful tool.*

*Really good work, thank you.*

*Well done and I hope it helps many people along the way.*

Users commented on the presentation of app content and features. Overall, they appreciated the mixture of audio, visual, or text within the different in-app activities and liked having a variety of activities and interactive components:

*For me it was especially helpful the components with video and audio. I do not like the components with reading as I am trying to minimise my screen time.*

*It’s very interactive which is a great feature to get focus back on track*.

In terms of the usefulness of specific therapeutic elements within the app, the relaxation practices, such as breathing or meditation, were particularly well received. Users indicated that these activities assisted them with stress reduction and anxiety relief, and experienced these activities to be beneficial for their overall well-being and joy:

*This a great tool for anyone who is suffering any kind of anxiety and doesn’t feel they can approach anyone with their problems*.

With respect to the thematic analysis, we further classified feedback into three categories: (1) qualities or features users liked, (2) features requested or missed by users, and (3) weaknesses of the app identified by users. These 3 categories helped us further understand how each component of the app helped (or could be improved) to meet user needs and promote adherence and acceptability.

Several app users (7/33, 21%) indicated they liked the daily check-in feature; it was suggested that this feature was very helpful for self-management of symptoms:

*I liked the daily check-in and to see how my two selected categories were tracked; mood and sleep. It was interesting to see the correlation between the two.*

*I like how the app asked how you are currently feeling, and then displayed content relevant to your feelings that you could do within the app*.

Around a quarter of users (8/33, 24%) provided suggestions to further improve the app, referring to specific app features; participants emphasized the importance of using “real-life” characters (ie, characters with lived experience) throughout the app, and more audio and visual elements to stimulate engagement with the app. Accurate presentation of time estimates for each of the activities was also recommended. The main weakness of the app, identified by users (8/33, 24%), related to difficulties navigating the app:

*Too many steps to find results.*

*Not very much direction in how to go through the app.*

*Was a little scattered to navigate*.

Others (5/33, 15%) highlighted problems with user engagement:

*Sometimes getting back into a repeat activity was tedious.*

*Maybe a little too simple, I tended to forget about using it.*

*Could be more interactive.*

*The repetitive nature of all the activities. It got a bit boring and started feeling less impactful.*

### Qualitative Interviews

In total, 8 out of the 33 participants (24%) who completed both the baseline and follow-up assessment agreed to participate in a semistructured interview. During the qualitative interviews, participants were asked to reflect on their experiences with the app and provide more detailed feedback on certain features of the app and its overall design and functionality. Participants felt that the app was very easy to use and provided relevant information for emergency service workers who are feeling overwhelmed and need some help managing stress or anxiety:

*I thought the app content was well balanced and covered a whole range of different issues and problems that people like me might helpful*

*For everyone who has a stressful job or stressful home life or school life or just life in general I think the app is really relevant, it’s really suitable for pretty much everybody across the board in our communities.*

*Of great use for first responders and community members who don’t have access to the mental health resources of the agencies*.

Participants also commented on the quality and credibility of the app, referring to the content as being of high standard, easy to use, and very educational. Participants furthermore felt that the app increased their awareness of MH issues not only within themselves, but also regarding work colleagues or employees:

*I highly recommend using the app for its insight into mood tracking. It showed me the relationship between anxiety and sleeping and the effect that it has a mental health when you don’t know unless you track it.*

*It makes it easier to start that conversation when thinking about suicide, gives you some ideas how to start that conversation:*

*I’m not feeling so great, Tom, can I chat with you*.

Participants suggested some minor design and content changes:

*There were too many layers. There were too many pages, sometimes that confused me.*

Participants recommended including additional reminders and adjunct components to help users stay engaged with the app:

*Maybe come up with a reminder, on your phone saying don’t forget to finish this. You know that you started yesterday or the day before just something like that*.

Further suggestions for improvement by emergency service workers app users were therefore largely focused on elements of user interface and user experience, such as a more intuitive home screen and changes to the background color within the app to reduce confusion around navigation and increase clarity in the presentation of the different activities.

### Symptom Change

Table 3 presents the means of the outcomes at both baseline and 1-month follow-up. Overall, at 1-month follow-up, there was a nonsignificant trend for improvement in general distress (*t*_32_=0.65; *P*=.52), depressive symptoms (*t*_32_=0.75; *P*=.46), and anxiety symptoms (*t*_32_=1.08; *P*=.29). This same nonsignificant trend was present in participants’ ability to manage traumatic stress (*t*_32_=−0.27; *P*=.79). Effect size estimates from baseline to 1-month follow-up were ≤0.2 for all outcomes.

**Table 3.** Changes in general distress, depression, anxiety, well-being, and traumatic stress coping outcome scores: mean difference, effect sizes, and standardized mean differences from baseline to 1-month follow-up.

	Baseline, mean (SD)	Postassessment^a^, mean (SD)	*t* test (*df*)	*P* value	Effect size (95% CI)	Standardized mean difference
K10^b^ (general distress)	18.67 (6.63)	18.18 (5.78)	0.65 (32)	.52	0.08 (−0.41 to 0.56)	0.48
PHQ-9^c^ (depression)	5.52 (4.14)	5.12 (3.98)	0.75 (32)	.46	0.10 (−0.39 to 0.58)	0.39
GAD-7^d^ (anxiety)	5.21 (4.38)	4.49 (4.20)	1.08 (32)	.29	0.20 (−0.25 to 0.58)	0.87
WHO-5^e^ (well-being)	56.97 (21.89)	56.12 (24.67)	0.28 (32)	.78	−0.06 (−0.55 to 0.42)	−1.55
CSE-T^f^ (traumatic stress coping)	47.82 (11.07)	48.12 (11.60)	−0.27 (32)	.79	−0.12 (−0.60 to 0.37)	−1.33

a Postassessment administered 1 month after baseline assessment.

b K10: Kessler Psychological Distress Scale.

c PHQ-9: 9-item Patient Health Questionnaire.

d GAD-7: 7-item Generalized Anxiety Disorder.

e WHO-5: 5-item World Health Organization Well-Being Index.

f CSE-T: Trauma Coping Self-Efficacy Scale.

## Discussion

### Principal Findings

The study evaluated the usability, feasibility, acceptability, and preliminary effectiveness of Build Back Better, an app-based MH intervention aimed at enhancing emotional well-being and positive MH among emergency service workers and volunteers.

The results from the postassessment surveys, in-app feedback, and qualitative interviews indicated that the Build Back Better app was found to have satisfactory levels of usability and acceptability. Participants indicated that the app content was relevant for emergency service workers and felt it was beneficial in increasing their capacity to manage stress and anxiety and in improving their general well-being. Users also appreciated the variety of features, functionality, and content within the app. The overall rating of the app was high to very high, with the majority of respondents indicating the app was easy to use, appropriate, and recommendable to others. These findings suggest that the app and its components are likely to meet emergency service workers’ expectations and requirements. However, qualitative analyses provided a more nuanced understanding of the perceived acceptability and usability of the app. Despite positive feedback on the user experience of the app, navigation concerns were raised that require design changes to enhance user experience and flow within the app. In addition, although most respondents understood the app content mostly or completely, a sizable proportion reported they only “somewhat” understood the content. Coupled with these navigation issues, both self-report feedback and objective app data highlighted issues pertaining to engagement and functionality. Not only was the mean total number of activities accessed within the app flow, but also questionnaire data suggested that half of the respondents found the app at best “somewhat” engaging. The 51% dropout rate for postsurvey completion may also be indicative of a lack of engagement with the app. Even though it is well-established that interventions delivered via MH apps are susceptible to low levels of engagement and poor adherence by their users [37-39], this can have implications for the effectiveness of the intervention delivered [40-42].

A secondary goal of this study was to determine the preliminary effectiveness of the Build Back Better app in reducing general distress and supporting (mental) well-being of emergency service workers. Although encouraging trends toward improvement in most symptom and well-being outcomes were found, these failed to meet significance. This raises vital questions that need to be addressed before larger testing. Considering the overall low symptom levels at baseline, floor effects are a possible implication of this lack of significance. However, as mentioned above, high rates of trial attrition left the trial underpowered, which would have also contributed to the risk of type 2 error. Furthermore, a related factor that requires attention is low program adherence. Despite the short length of the trial and wide variety of available exercises within the app, engagement was low, with the app being used, on average, 5 to 6 times for the duration of the 1-month trial. Surprisingly, adherence was considerably lower than that observed in previous trials of similar apps [18,24,35,36]. This raises questions about both the app content and the free-form structure of the app. Compared to previous trials in which the app content was delivered in a linear fashion (introducing one new activity at a time), users of this app had greater choice and were able to access recommended content on demand. Generally, such an individualized approach using personally tailored content is viewed as a positive means to enhance engagement in digital interventions [43,44], but in this study, it may have left users feeling overwhelmed and undirected, as from the qualitative data findings, some participants reported feeling overwhelmed or confused by the lack of direction in how to navigate the app content. Previous research has shown that providing users with more choices and actions leads to increased cognitive strain, and this may negatively affect acceptability [41,45,46]. Further research should investigate which activities users prefer to do without those activities becoming burdensome or overwhelming, while also creating an app that allows independence and control.

### Limitations

There are several limitations of this study, which should also be acknowledged. Emergency service workers are an at-risk population due to high rates of trauma exposure and related illness [47]. Although the self-reported ability of participants to manage traumatic stress was captured for the duration of the trial, trauma exposure or levels of traumatic distress were not examined in the current sample, and there were no restrictions on eligibility related to these factors. Overall, the sample reported very few symptoms of MH illness. Interestingly, despite low baseline levels of anxiety, depression, and psychological distress, most participants also scored in the lower percentile of well-being scores. Regardless, not only did this potentially impact capacity for symptom change, but it also limited comparison of outcomes based on severity. Such a program is likely to have a differing effect on individuals experiencing differing levels of psychological well-being at baseline, possibly because there are considerable between-individual differences in the response to these interventions [48,49]. The Build Back Better app itself was designed as an MH promotion and selective prevention app, directed toward at-risk groups or individuals with physical, psychological, or social risk factors associated with mental illness development, but this study lacked the power to adequately test this.

Another limitation of this study was the high attrition rate, with nearly half of the participants failing to complete their follow-up assessments after 30 days. Although high attrition is common in unguided trials [50-52], nonrespondents in this study may have been affected by digital access issues. Of the study sample, 34.3% lived in regional or remote areas, and these rural residents are still less likely than those living in suburban areas to have home broadband and could have faced challenges to accessing the internet and completing their follow-up surveys. While the Build Back Better app was designed to allow the user to run the app in different conditions and locations, a lack of internet connectivity could have prevented users from completing the online follow-up surveys via Black Dog Institute’s Online Research Platform.

### Future Directions

Further investigation is needed to determine the potential protective benefits of the Build Back Better app under more controlled conditions, in light of current knowledge on between-individual differences in how individuals respond to interventions. In addition to a larger, appropriately powered controlled trial, future evaluation should consider baseline cut-offs for symptom severity, which may influence the effectiveness of the app. This study used a heterogeneous sample of emergency service workers, which may have impacted findings. While this diversity was seen as a strength of the study, and the app was designed to cater to a range of emergency service workers, there is the potential that the content may have been received differently across professions. This suggests that a more tailored version of the app, designed specifically for different professions or roles, could improve engagement and content relevance.

We recognize that the lower participation rate in the follow-up questionnaires and qualitative interviews is a limitation of this study. Although the interviews were structured with thoughtful, targeted questions designed to elicit rich, in-depth responses, the low response rate limits the representativeness and diversity of the perspectives gathered. Similarly, the high dropout rate from the follow-up assessments reduces the robustness of our conclusions and the generalizability of our findings. Further analysis comparing postcompleters and noncompleters revealed that the only significant difference between these groups was gender, with males being less likely to complete the postassessment. Dropout was not strongly associated with baseline symptom severity or well-being; however, additional research with a larger sample size would help clarify these patterns and improve the reliability of the findings.

The high attrition rate, furthermore, highlights the need for future studies to address potential barriers to engagement, such as digital access issues. Identifying and mitigating these barriers through improved app design or more effective follow-up communication strategies could provide more reliable data on the effectiveness of mobile phone–based health initiative interventions in high-risk populations. For example, testing different engagement strategies, such as varying the frequency of reminders or using multiple forms of communication (eg, SMS text messages and phone calls), could help improve follow-up rates. The protocol for this trial involved 2 email reminders for follow-up assessment. In future evaluations of MH apps, consideration is needed regarding other reminder options. SMS text message reminders and phone calls might be a more reliable form of contact for this group, considering their location and the nature of many emergency service workers’ work. Moreover, varying frequency of email reminders and varying content could help engage participants [53]. Nevertheless, once recruited, participants seemed motivated to start using the app, and the 94% (67/71) baseline completion rate was strong.

Finally, feedback from users in this pilot study revealed several areas for improvement in the app to increase usability and engagement. Participants in this study identified issues particularly around app navigation and user interface, that is, clearer design, short interactions with the app, easy access, but the app users also provided suggestions for improving user engagement, for example, via incorporating reminders to engage with the app, and involving those with lived experience in the activities within the app. This feedback is consistent with observations from reviews of efficacy studies on smartphone interventions, in which apps incorporating more elements aimed at promoting user engagement have shown larger effect sizes on several MH outcomes [42]. Proposed design changes therefore include (1) improved navigation via refined home screen emphasizing the presentation of activities in modules, (2) a more visually engaging home screen, and (3) increased accessibility and understanding of the in-app search function via clearer instructions and use of different design elements. In addition, future marketing and promotion efforts should emphasize involving those with lived experience to strengthen the app’s relevance and appeal. Finally, this study furthermore highlighted the need for more nuanced app usage data to further explore use patterns.

### Conclusions

The findings of this pilot study support the usability and acceptability of the Build Back Better app among emergency service workers, complemented by valuable user input that will inform modification of the app design to further enhance the in-app user experience. The app was found to have satisfactory levels of engagement, usability, feasibility, and acceptability. However, evidence of change in MH outcomes could not be gained from this small pilot study. Comparison of the Build Back Better app with a control condition in a larger trial will help to address the limitations of this study and further elucidate (clinical) MH outcome findings. To further advance the field in digital MH options, it is furthermore important for future research to explore which engagement metrics (log-ins, activities completed, time spent in app, etc) could have an impact on improving MH outcomes.

## Abbreviations

CSE-T: Trauma Coping Self-Efficacy Scale
*DSM-IV*: *Diagnostic and Statistical Manual of Mental Disorders, Fourth Edition*
GAD-7: 7-item Generalized Anxiety Disorder
K10: Kessler Psychological Distress Scale
MARS: Mobile Application Rating Scale
MH: mental health
PHQ-9: 9-item Patient Health Questionnaire
PTSD: posttraumatic stress disorder
RCT: randomized controlled trial
WHO-5: 5-item World Health Organization Well-Being Index
